# Digital Frequency Customized Relieving Sound for Chronic Subjective Tinnitus Management: Prospective Controlled Study

**DOI:** 10.2196/60150

**Published:** 2025-01-17

**Authors:** Dongmei Tang, Yuzhu Peng, Dantong Gu, Yongzhen Wu, Huawei Li

**Affiliations:** 1 ENT Institute and Department of Otorhinolaryngology Eye & ENT Hospital Fudan University Shanghai China; 2 NHC Key Laboratory of Hearing Medicine Research Shanghai China; 3 Shanghai Zehnit Medical Technology Co Ltd Shanghai China; 4 Clinical Research Unit of Eye & ENT Hospital Fudan University Shanghai China

**Keywords:** tinnitus, digital frequency customized relieving sound, unmodified music, sound therapy, prospective study, mobile phone

## Abstract

**Background:**

Tinnitus is a major health issue, but currently no tinnitus elimination treatments exist for chronic subjective tinnitus. Acoustic therapy, especially personalized acoustic therapy, plays an increasingly important role in tinnitus treatment. With the application of smartphones, personalized acoustic stimulation combined with smartphone apps will be more conducive to the individualized treatment and management of patients with tinnitus.

**Objective:**

The aim of this study was to evaluate the efficacy of a new personalized approach known as the digital frequency customized relieving sound (DFCRS) for tinnitus treatment and to explore the factors that may influence its therapeutic effect.

**Methods:**

Patients with subjective tinnitus were enrolled in this study from July 14, 2020, to May 24, 2021, in the tinnitus specialist clinic of Eye and ENT Hospital, Fudan University, Shanghai, China. In this nonrandomized concurrent controlled trial, a total of 107 participants were assigned to listen to personalized DFCRS through our developed app, while the other 77 participants who did not want to download and use the app were assigned to listen to unmodified music (UM). All the recruits were instructed to listen to DFCRS or UM for at least 2 hours a day and complete follow-up assessments at baseline, 1, 2, and 3 months. Multidimensional assessment scales, that is, Tinnitus Handicapped Inventory (THI), Hospital Anxiety and Depression Scale (HADS), Athens Insomnia Scale (AIS), Fear of Tinnitus Questionnaire (FTQ), and Tinnitus Catastrophizing Scale (TCS) were used to evaluate the severity of tinnitus and the quality of life. Linear mixed models were used to test for changes in the THI scores across 3 months of acoustic treatment between group (DFCRS or UM treatment) and time. A multiclass logistic model was built with a stepwise function to determine the influence of the different covariates on the effects of acoustic treatment.

**Results:**

The results of the multidimensional assessment scales after 3 months of treatment showed that DFCRS-treated patients had significant tinnitus relief compared to those in the UM group. Linear mixed models revealed a significant reduction in the THI scores over time (*P*<.001), with the DFCRS group showing significantly greater improvement than the UM group (*P*<.001). At 3 months, 92.5% (99/107) of the patients undergoing DFCRS reported tinnitus relief or disappearance, and longer daily treatment time was associated with better outcomes (*P*=.007). Multiclass logistic regression confirmed that longer treatment time (odds ratio [OR] 13.07-64.78; *P*<.001) and more severe tinnitus at baseline (OR 10.46-83.71; *P*<.001) predicted better treatment response. All secondary outcomes (HADS, AIS, FTQ, TCS) showed significant improvements over time (*P*<.001).

**Conclusions:**

Our study suggests that DFCRS is a new promising and noninvasive therapy for chronic tinnitus, and it can be delivered through a mobile app to bring more convenience to patients with tinnitus.

## Introduction

Tinnitus, a common concern in the otolaryngology clinic, is described as the perception of sound without a corresponding external stimulus [1]. People can experience tinnitus constantly or intermittently from a single ear, both ears, and within the head. The presence of tinnitus can be annoying, affecting the patients’ overall quality of life (QoL) and even negatively impacting their mental health [2]. Epidemiological studies have shown that tinnitus affects approximately 10%-25% of the adults [3,4], and tinnitus-induced anxiety, irritability, depression, sleep disturbances, and concentration disorders seriously threaten the cognitive and neurological health of approximately 1% of the adults [5]. Therefore, the search for an efficient treatment for tinnitus is urgently needed.

To date, there is a lack of approved medications that can eliminate tinnitus or provide a completely satisfactory cure [6,7]. However, there are several intervention strategies that can help patients cope with tinnitus. In recent decades, sound therapy has been broadly applied in exploring clinical treatments for tinnitus [8-12]. According to the sound generation method, sound therapy can be divided into 2 main types: unmodulated and modulated sound therapy [13]. Tinnitus masking and tinnitus retraining therapy are the 2 most commonly used unmodulated strategies, and their ultimate goal is to help patients with tinnitus become accustomed to tinnitus, attenuating excessive attention to it and thus reducing the tinnitus-related negative emotions [14-17]. Although some findings have confirmed their effectiveness in improving the QoL of patients with tinnitus [18], a randomized clinical trial showed no significant difference in the outcomes between tinnitus retraining therapy and placebo-sound treatment control groups [19]. Additionally, the current unmodulated sound therapy does not appear to change the loudness of tinnitus, limiting the possibility of tinnitus elimination [12].

Conversely, customized sound therapy is designed to encourage the reorganization of abnormal neural connections or to inhibit pathological neural activity, which is more promising in effectively eliminating tinnitus [10,20]. Current research has confirmed the superior effectiveness of customized sound therapy over unmodulated therapy [21]. For example, neuromonics tinnitus treatment involves adding or excluding individual broadband frequency sounds to relaxing music to produce intermittent tinnitus perception and facilitate desensitization to tinnitus [22-24]. It is worth noting that tailor-made notched music training [10] and tinnitus pitch-matched therapy [11] used 2 opposite sound-processing strategies; however, both produced positive results [25-27]. Thus, the underlying mechanisms of tinnitus alleviation, which might be linked to the pathogenesis of tinnitus, should be elucidated in the future. Although various sound therapies differ in strategies and effectiveness, there is a consensus that the effects of sound therapy are not immediate and need to be sustained over a period of time. We reviewed the follow-up time of different acoustic treatments [28]. For short ones such as the Heidelberg music therapy, the follow-up time can be 5 days, 1 week, or 3 weeks. In interventions such as tailor-made notched music, pitch-matched therapy, and tinnitus masking, the follow-up time can be 1 month to 3 months. Tinnitus retraining therapy, neuromonics tinnitus treatment, acoustic coordinated reset neuromodulation therapy, tailor-made notched music, and many other studies have a long follow-up time—more than 6 months or even 12 months, 18 months, 36 months, and so on.

To date, the mechanism of subjective tinnitus has not been fully elucidated. Generally, peripheral hearing loss may be the trigger of subjective tinnitus [12,29]. Due to auditory deprivation, the activity of the affected peripheral auditory nerves is reduced, and neuroplasticity directs adaptive changes in the central auditory system when the brain loses input from the ear [30,31]. Based on this, we designed a new personalized tinnitus relief model known as the digital frequency customized relieving sound (DFCRS), which generates 2 surrounding narrow bands (each 1/3 octave) according to the individual’s matched tinnitus frequency and then dynamically enhances the intensity of the 2 designated bands by 10 dB on soft and relieving music [32]. With the popularity of mobile smart devices and the improved stability and convenience of mobile apps, which are increasingly being used in the sound therapy of patients with tinnitus [33,34], we developed an app named the Fudan Tinnitus Relieving System (FTRS) [35,36] to run this model for monitoring, management, and follow-up evaluation of tinnitus treatment. In this study, we aimed to investigate the effectiveness of DFCRS by using a self-designed FTRS app compared to that of unmodified music (UM) for treating tinnitus. Furthermore, we explored the possible factors that might influence the treatment effectiveness of DFCRS.

## Methods

### Study Design

Patients with subjective tinnitus were enrolled from the tinnitus specialist clinic of Eye & ENT Hospital of Fudan University from July 14, 2020, to May 24, 2021. All the included participants were Mandarin speakers, aged over 18 years, and able to communicate with instructors without any hearing aids. The average pure tone threshold (0.5, 1, 2, 4 kHz) should be no more than 55 dB HL of the worse ear. The participants were required to understand the purpose and process of the study, complete at least 3 follow-up visits in the clinic, and sign the informed consent prior to the commencement of the experiment. Patients with pulsatile tinnitus, objective tinnitus, or clear primary causes such as otosclerosis, Meniere disease, and acute stage of sudden sensorineural hearing loss were excluded from this study. Moreover, for safety reasons, patients with severe health issues such as cardiovascular and cerebrovascular diseases or tumors were excluded from this study. This was a nonrandomized concurrent controlled trial, and we followed the TREND (Transparent Reporting of Evaluations with Nonrandomized Designs) guidelines for nonrandomized controlled trials (Multimedia Appendix 1). A total of 107 eligible patients who tended to use the FTRS app for acoustic therapy received DFCRS, and they were labeled as the experimental group. The other 77 patients who refused to use the app received UM, serving as the control group. The experimental group and the control group were recruited and provided acoustic therapy at the same time. All participants completed 4-timepoint follow-up questionnaires.

### Ethics Approval

All tests were approved by the institutional review board of Eye & ENT Hospital of Fudan University (2017048). All patients signed the informed consent forms, and sensitive information relevant to patient privacy was removed before the analysis. Both forms of acoustic therapy did not add any cost to the patient, and each patient could also receive an appointment or a plus number service for the tinnitus specialist clinic.

### Patient Assessment

The baseline data were collected mainly from 4 aspects: general information, characteristics of tinnitus, audiological and tinnitus assessments, and results of the multidimensional assessment scales of tinnitus. General information included details on age, gender, medical history, and treatment history. The characteristics of tinnitus included information on position, course, pitch, and correlative factors of tinnitus. Audiological and tinnitus tests included pure-tone audiometry (125 Hz-8 kHz), acoustic immittance, and tinnitus pitch matching. Five standardized questionnaires in the Chinese version were used to evaluate the severity of tinnitus and the QoL of patients with tinnitus from multiple dimensions.

#### Tinnitus Handicap Inventory

The Tinnitus Handicap Inventory (THI) is a self-report measure first developed in 1996 [37]. It contains 25 items with 0, 2, and 4 points each to quantify the functional, emotional, and catastrophic feedback to tinnitus. The total number of points is up to 100, with a higher score indicating more severe tinnitus-related discomfort and disability.

#### Hospital Anxiety and Depression Scale

The Hospital Anxiety and Depression Scale (HADS) consists of 14 items, 7 of which belong to the subscale for anxiety (HADS-A) and the other 7 questions indicate the subscale for depression (HADS-D) [38]. Due to the close correlation between tinnitus severity and psychiatric disorders, HADS was introduced to patients with tinnitus to identify emotional disturbances that reflect therapeutic efficacy [39].

#### Athens Insomnia Scale

The Athens Insomnia Scale (AIS) is commonly used to measure sleep quality [40]. It includes 8 items with a total score ranging from 0 (no sleep disorder at all) to 24 (the most severe insomnia). Changes in sleep disorders—one of the most common concomitant symptoms of tinnitus—can be used to evaluate the effects of tinnitus treatment.

#### Fear of Tinnitus Questionnaire

The Fear of Tinnitus Questionnaire (FTQ) is a self-report measure containing 17 items, with 1 point each, which intends to assess the levels of fear of tinnitus [41]. Higher scores indicate a greater fear of tinnitus. Researchers can estimate tinnitus status based on patients’ attitudes and moods toward tinnitus.

#### Tinnitus Catastrophizing Scale

The Tinnitus Catastrophizing Scale (TCS) is a 13-item measure developed to assess how catastrophizing respondents are regarding tinnitus [42]. The instrument is rated on a 5-point scale from 0 to 4, with higher scores indicating higher states of tinnitus-specific distress.

#### Tinnitus Severity Assessment

The Tinnitus Severity Assessment scale divides patients with tinnitus into 4 grades according to subjective tinnitus-related impairment in daily life [2]. Patients with grade I or no impairment are not annoyed by tinnitus, whereas patients who are unable to work or live normally will be confirmed as having grade IV severe impairment.

### Acoustic Therapy Procedure

In the DFCRS-treated group, according to precise audiological and tinnitus tests, a series of 4 endpoints of the modulation range adjacent to the tinnitus pitch were calculated using our specific algorithm (Multimedia Appendix 2). Based on these 4 endpoints, 2 specific frequency bands of 1/3 octave intervals below and above the tinnitus pitch were confirmed. Thereafter, with the assistance of the FTRS app, soft and relieving music was processed by dynamically increasing the intensity of both specified bands with a 10-dB gain, thus outputting the personalized relieving sound for tinnitus. Accordingly, in the UM-treated group, the unmodified original music was used.

After baseline data completion at the initial point in time (T0), patients in the DFCRS-treated group were trained to use the FTRS app correctly, while others in the UM-treated group were instructed to listen to UM through their favorite players. All participants were required to listen to the modified or unmodified sound in a quiet environment with a comfortable volume to avoid possible hearing damage.

In this study, the participants were not allowed to receive any other simultaneous treatments that might interfere with sound therapy. To obtain a better effect, all patients were encouraged to be exposed for as much time as possible in the therapeutic sound environment, and the recommended duration was at least 2 h/day. In addition, the participants were instructed to record the time of daily sound exposure accurately and truthfully. Finally, researchers maintained contact with the participants through various accessible means such as phone calls, WeChat, emails, and tinnitus specialist clinic visits.

### Tinnitus Counseling and Education

Well-trained otologists implemented counseling and education for all patients with tinnitus. The main topics included the etiology and basic pathogenesis of tinnitus, the relationship between tinnitus and hearing loss, the interpretation of audiological and tinnitus test results, the psychoacoustic characteristics of tinnitus, the relationship between tinnitus and attention, guidance on exploring common methods to divert attention, and relaxation training. During the entire procedure, the participants’ confusion and discomfort with regard to tinnitus were patiently, promptly, and sufficiently dispelled.

### Outcome Measures

There were 4 timepoints in total, including the baseline when the participants were enrolled and at 1, 2, and 3 months after commencing treatment. The primary outcome was the THI scores from baseline to follow-up endpoints. In addition, the other assessment instruments (HADS, AIS, TCS, and FTQ) as secondary outcomes also provided longitudinal insights into the efficacy of tinnitus treatment.

### Statistical Analysis

Statistical analyses were performed using R software (version 4.1.2; R Foundation for Statistical Computing) with the lme4, nnet, and ggpubr packages. Two-sided values of *P*<.05 were deemed statistically significant. Demographics and baseline characteristics such as age, sex, tinnitus course, location, tinnitus pitch, severity, and assessment instruments (THI, HADS-A, HADS-D, AIS, FTQ, and TCS) were summarized by groups that were defined by the treatment results. We summarized the descriptive statistics of the continuous variables (mean, standard deviation or median, and quartile) and categorical variables (number and proportion of participants in each category). Differences in the characteristics among the groups were tested with chi-square for categorical variables; 1-way analysis of variance was used for normally distributed continuous variables, and the Kruskal–Wallis test was used for nonnormal continuous variables. A 2-tailed *t* test was applied to compare the mean multidimensional assessment data before and after treatment and to determine the differences between subgroups. Linear mixed models were used to test for changes in the THI scores across 3 months of acoustic treatment between groups (DFCRS or UM treatment) and time. A multiclass logistic model was built with a stepwise function to determine the influence of different covariates on the effect of acoustic treatment, and Akaike information criterion/Bayesian information criterion/corrected Akaike information criterion was used to select the best model.

## Results

### Demographic and Tinnitus Characteristics of the Participants at Baseline

A total of 184 patients with tinnitus (107 in the DFCRS treatment group and 77 in the UM control group) completed the baseline data collection and 3 follow-up visits. After the 3-month treatment, patients were asked to report the effectiveness of tinnitus treatment based on their subjective perceptions. The baseline data are shown in Table 1. In the DFCRS treatment group, 50.5% (54/107) were men and 49.5% (53/107) were women, aged 45 (range 35.5-57) years, with a tinnitus duration averaged at 12 (range 4-24) months. In the UM group, 55% (42/77) were males, the age was 50 (range 37-59) years, and the tinnitus duration was 8 (range 3-42) months. The patients’ gender and age did not differ significantly between the experimental group (DFCRS) and the control group (UM). We also compared other baseline data and observed no significant differences in the distribution of tinnitus characteristics involving location, frequency, and severity assessed by multidimensional tinnitus scales (HADS, AIS, Visual Analog Scale, FTQ, and TCS) between the 2 groups. Besides, there were no significant differences in the hearing levels in either ears between the 2 groups. However, the THI scores differed at baseline between the 2 groups (UM group: median 40, range 24-60; DFCRS group: median 48, range 33-66; *P*=.01). The overall baseline data were comparable between the DFCRS group and the UM group, suggesting that the following therapeutic effect was not significantly influenced by pre-experimental demographic information and characteristics of tinnitus.

**Table 1:** Demographic and tinnitus characteristics of the participants in the digital frequency customized relieving sound–treated group and the unmodified music control group at baseline.

		Unmodified music (n=77)	Digital frequency customized relieving sound (n=107)	*P* value	
**Gender, n (%)**					.69
	Male	42 (55)	54 (51)		
	Female	35 (45)	53 (50)		
Age (years), median (IQR)		50 (37-59)	45 (35.50-57)	.39	
Tinnitus course (months), median (IQR)		8 (3-42)	12 (3-24)	.88	
**Location of tinnitus, n (%)**					.31
	Unilateral	37 (48)	40 (37)		
	In head	3 (4)	7 (7)		
	Bilateral	37 (48)	60 (56)		
Tinnitus Handicapped Inventory, median (IQR)		40 (24-60)	48 (33-66)	.01	
Hospital Anxiety and Depression Scale-subscale anxiety, median (IQR)		5 (2-7)	5 (2-8)	.40	
Hospital Anxiety and Depression Scale-subscale depression, median (IQR)		4 (1-7)	4 (2-7)	.18	
Athens Insomnia Scale, median (IQR)		6 (4-9)	7 (4-10)	.11	
Visual Analog Scale, median (IQR)		5 (3-6)	5 (4-6)	.05	
Fear of Tinnitus Questionnaire, median (IQR)		6 (4-10)	7 (5-10)	.26	
Tinnitus Catastrophizing Scale, mean (SD)		22.97 (10.42)	24.38 (9.89)	.36	
Left ear pitch, median (IQR)		3642.50 (1933.75-5780)	7175 (6350-8000)	.07	
Right ear pitch, median (IQR)		6350 (4435-6350)	6350 (4260-8000)	.47	
Pure tone average-right^a^, median (IQR)		16.67 (15-21.67)	16.67 (15-20.42)	.55	
Pure tone average-left^a^, median (IQR)		16.67 (15-23.33)	17.50 (15-23.75)	.88	

^a^Average air conducted pure tone hearing thresholds of 500, 1000, 2000, and 4000 Hz.

### Mixed Effect Model Indicated Effectiveness of DFCRS Treatment

As the primary outcome, the THI scores were calculated using the linear mixed model in both DFCRS and UM treatment groups. The results of the linear mixed model revealed significant main effects of follow-up time (estimate=–0.119; *P*<.001) (Table 2), which showed a general decrease in the THI scores over the trial duration. Besides, there were significant effects in the DFCRS group compared to those in the control group, and the negative value revealed a remarkable treatment effectiveness of DFCRS measured by THI compared to that of UM in the control group (estimate=–16.6469; *P*<.001).

**Figure 1:**
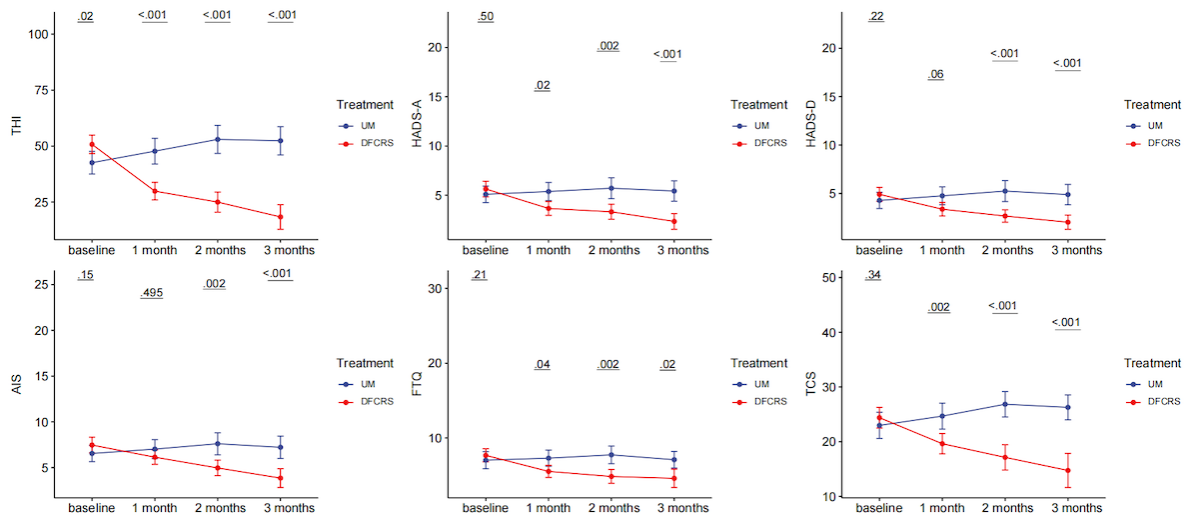
Comparison of the multidimensional assessment scales between the digital frequency customized relieving sound treatment group and the unmodified music control group at 4 timepoints. Data are expressed as median (IQR). AIS: Athens Insomnia Scale; DFCRS: digital frequency customized relieving sound; FTQ: Fear of Tinnitus Questionnaire; HADS-A: Hospital Anxiety and Depression Scale-subscale anxiety; HADS-D: Hospital Anxiety and Depression Scale-subscale depression; TCS: Tinnitus Catastrophizing Scale; THI: Tinnitus Handicapped Inventory; UM: unmodified music.

Apart from the follow-up time and treatment procedure, no other factors were found to contribute to a significant effect in the linear mixed model. Comparisons of the multidimensional assessment scales between the DFCRS-treated group and the UM control group at 4 timepoints are shown in Figure 1. During the 3 months of treatment, almost all of the scores on the 6 scales in the DFCRS-treated group showed significant decline compared to those in the UM control group, indicating better effectiveness of DFCRS in managing tinnitus. We found that UM had no significant therapeutic effect on tinnitus (Table S1 in Multimedia Appendix 3). Moreover, the differences were mostly maintained or were even enlarged across time especially after 1 month of treatment. No adverse effects were reported during follow-up in both groups.

**Table 2:** Linear mixed effects on tinnitus relief after digital frequency customized relieving sound or unmodified music treatment.

		Estimate	SE	*t* test *(df)*	*P* value
(Intercept)		56.13335	6.032316	9.305439 (509)	<.001
Follow-up time		–0.119	0.015712	–7.57409 (509)	<.001
**Group**					
	Control	Reference	Reference	Reference	Reference
	Digital frequency customized relieving sound	–16.6469	3.114957	–5.34417 (173)	<.001
**Gender**					
	Male	Reference	Reference	Reference	Reference
	Female	3.57652	3.087018	1.158567 (173)	.25
Age		–0.10198	0.114023	–0.89438 (173)	.37
**Location**					
	Unilateral	Reference	Reference	Reference	Reference
	In the brain	–9.39239	6.990866	–1.34352 (173)	.18
	Bilateral	3.73601	3.248982	1.149901 (173)	.25
Course of tinnitus		–0.00097	0.03535	–0.02749 (173)	.98

### Multiclass Logistic Model Predicted Factors That Affect the Effectiveness of DFCRS Treatment for Tinnitus

**Figure 2:**
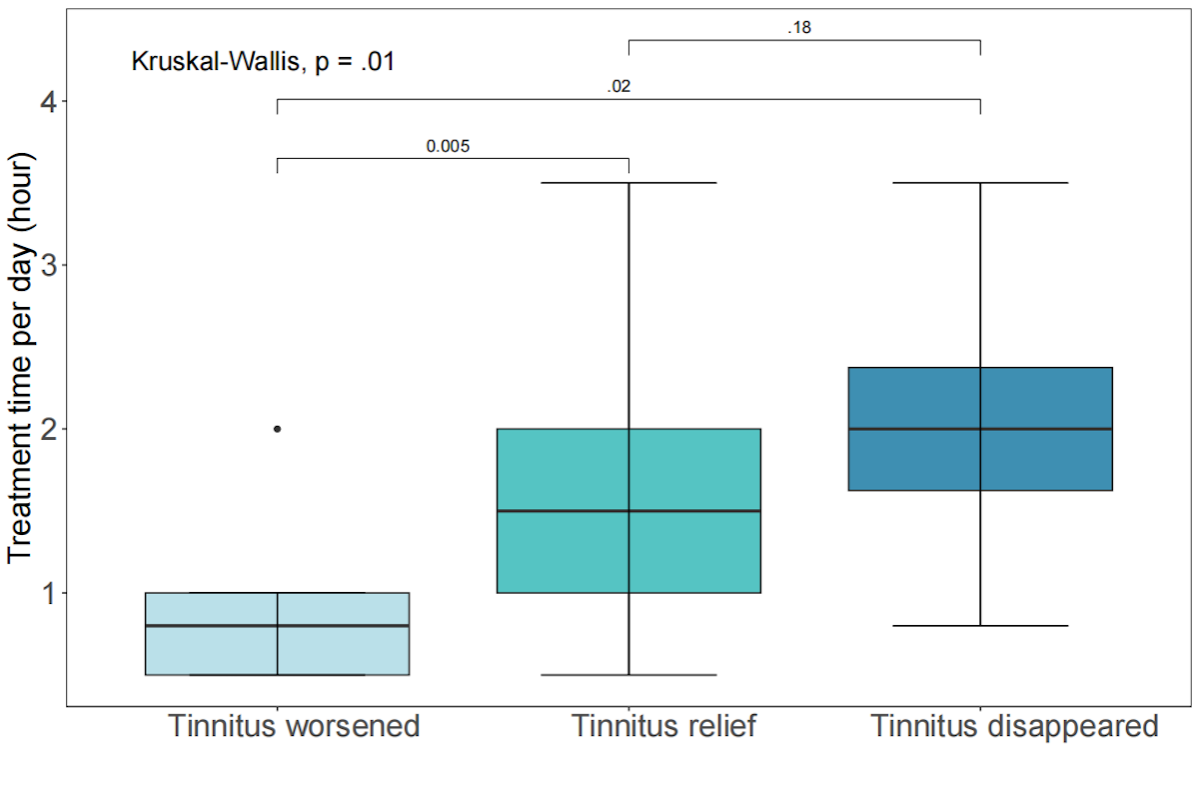
Differences in the treatment time of digital frequency customized relieving sound in the 3 treatment outcome groups according to subjective reports. Data are presented as median (IQR).

To further identify the factors affecting the effectiveness of DFCRS, we analyzed the statistical relationships among gender, age, acoustic treatment time per day, various tinnitus characteristics, and treatment results according to patients’ subjective reports. After 3 months of DFCRS treatment, 86.9% (93/107) of the patients reported tinnitus relief, 5.6% (6/107) reported that tinnitus disappeared, and 7.5% (8/107) reported worsened tinnitus (Table S2 in Multimedia Appendix 3). The patients’ gender and age did not differ significantly among the 3 different treatment outcome groups. There were no significant differences in the distribution of characteristic tinnitus indicators such as location, tone, course, and severity assessed by multidimensional tinnitus scales among the 3 subgroups as well. Interestingly, the data showed that there was a significant difference in the treatment time per day among the 3 groups (*P*=.007). Participants whose tinnitus was alleviated or disappeared received sound therapy for a longer time than those who reported worsening tinnitus, indicating a time-dose–dependent relationship for implementing DFCRS as a tinnitus treatment strategy (Table S2 in Multimedia Appendix 3). We then conducted a two-by-two comparison among the 3 subgroups, and the results revealed that significant differences existed between the worsening tinnitus group and the tinnitus relief group (*P*<.01) as well as the worsening tinnitus group and the tinnitus disappeared group (*P*<.05; Figure 2). This finding preliminarily confirmed that an increase in the exposure of DFCRS treatment contributed to better efficacy in managing tinnitus.

For more precise analysis, we built a multiclass logistic model to determine the influence of different covariates on acoustic treatment effects (Table 3). The results showed that compared to patients whose tinnitus worsened, patients who reported tinnitus relief had a longer treatment time per day (odds ratio [OR] 13.07, 95% CI 4.19-40.78; *P*<.001), and patients who reported tinnitus disappearance had the longest daily treatment time (OR 64.78, 95% CI 11.63-360.77; *P*<.001). In addition, patients with more severe tinnitus at baseline tended to respond better to acoustic treatment. More specifically, patients with severity III were more likely to have tinnitus relief (OR 40.37, 95% CI 3.42-477.14; *P*<.001) than patients whose tinnitus severity level was I. Similarly, patients with severity IV were more likely to have tinnitus relief (OR 10.46, 95% CI 2.12-51.62; *P*<.001) or tinnitus disappearance (OR 83.71, 95% CI 5.9-1188.28; *P*<.001) than those with tinnitus severity I.

**Table 3:** Multiclass logistic model results of the influence of different covariates on the customized acoustic treatment effect.

					Odds ratio (2.50% CI-97.50% CI)			*P* value
Worsening					Reference			Reference
**Relief from tinnitus**								
	(Intercept)			258.01 (25.56-2604.61)			<.001	
	Treatment time			13.07 (4.19-40.78)			<.001	
	**Gende**r							
		Male	Reference			Reference		
		Female	1.05 (0.33-3.31)			.94		
	Age			0.96 (0.92-1.01)			.08	
	Tinnitus course			1.01 (0.99-1.02)			.37	
	**Status**							
		Continued	Reference			Reference		
		Intermittent	1.42 (0.09-22.24)			.80		
	**Severity**							
		Ⅰ	Reference			Reference		
		Ⅱ	3.1 (0.75-12.9)			.12		
		Ⅲ	40.37 (3.42-477.14)			<.001		
		Ⅳ	10.46 (2.12-51.62)			<.001		
**Disappearing tinnitus**								
	(Intercept)			0.55 (0.01-49.61)			.79	
	Treatment time			64.78 (11.63-360.77)			<.001	
	**Gender**							
		Male	Reference			Reference		
		Female	0.28 (0.03-2.42)			.25		
	Age			0.94 (0.85-1.05)			.30	
	Tinnitus course			0.74 (0.5-1.09)			.13	
	**Status**							
		Continued	Reference			Reference		
		Intermittent	221.23 (4.75-10,304.32)			.01		
	**Severity**							
		Ⅰ	Reference			Reference		
		Ⅱ	7.11 (0.38-133.18)			.19		
		Ⅲ	20.42 (0.7-591.93)			.08		
		Ⅳ	83.71 (5.9-1188.28)			<.001		

### DFCRS Treatment Demonstrated a Dose-Dependent Effectiveness As Measured by Multidimensional Assessment Scales

**Figure 3:**
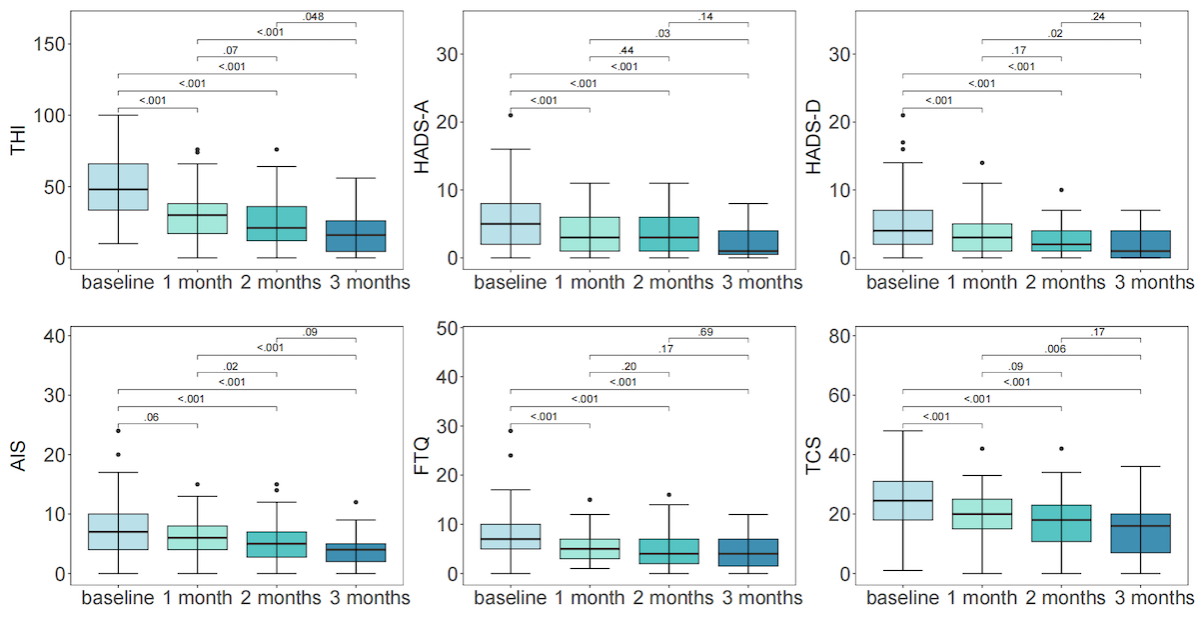
Box and whisker plots for changes in the scores of the multidimensional assessment scales in digital frequency customized relieving sound–treated patients at 4 timepoints. After 3 months of digital frequency customized relieving sound treatment, the scores on all the 6 scales were significantly lower than those at baseline. Data are presented as median (IQR). AIS: Athens Insomnia Scale; FTQ: Fear of Tinnitus Questionnaire; HADS-A: Hospital Anxiety and Depression Scale-subscale anxiety; HADS-D: Hospital Anxiety and Depression Scale-subscale depression; TCS: Tinnitus Catastrophizing Scale; THI: Tinnitus Handicapped Inventory.

After 3 months of DFCRS treatment, the scores of all the multidimensional scales significantly decreased compared with the baseline data (*P*<.001 for all scales), indicating the efficacy of DFCRS therapy in managing tinnitus. The THI score decreased from 48.00 (95% CI 32.00-66.00) points at baseline to 30.00 (95% CI 18.00-42.00) points at the 1-month follow-up, to 22.00 (95% CI 12.00-36.00) points at the 2-month follow-up, and finally to 18.00 (95% CI 8.00-30.00) points at the 3-month follow-up. Significant differences were observed between the scores at baseline and 3 consecutive months of follow-up (*P*<.001; Figure 3).

By comparing the 3 follow-up scores with the baseline of the other secondary outcomes, we observed that a more significant decline occurred in the following follow-up than in the preceding one (HADS-A, AIS, FTQ: *P*<.001 at 1-month follow-up, *P*<.001 at 2- and 3-month follow-ups; and HADS-D, TCS: *P*<.01 at 1-month follow-up, *P*<.001 at 2- and 3-month follow-ups). These significant differences suggested that there was a time-dependent increase in the effectiveness during the experimental procedure.

We also analyzed the differences between 2 of the 3 follow-up outcomes of the 6 assessment scales. Regarding THI scores, significant differences were observed between 2 and 3 months of follow-up (*P*<.05) and between 1 month and 3 months of follow-up (*P*<.001). In contrast, the THI scores did not decrease significantly from 1 month of follow-up to 2 months of follow-up (*P*=.07). A similar reduction was also found in the TCS scores (1 month vs 2 months: *P*=.09; 1 month vs 3 months: *P*<.01; 2 months vs 3 months: *P*=.17; baseline vs 3 months: *P*<.001), indicating that the therapeutic effect of DFCRS on tinnitus impairment and catastrophizing was observed at 2 months and was maintained in the third month.

## Discussion

Customized sound therapy is an advanced method for treating subjective tinnitus that can effectively reduce the loudness of tinnitus and alleviate its negative impact on patients [12]. In this clinical trial, we explored the therapeutic effect of a new self-developed acoustic treatment named DFCRS [32] compared to that of UM over 3 months. Based on the changes in the scores of multidimensional assessment scales and patients’ subjective judgments of symptom change, we concluded that the DFCRS delivered by our developed FTRS app [36] was more effective than UM in relieving subjective tinnitus and improving the QoL of patients with tinnitus. Moreover, better effects were obtained as the treatment course was extended or if the patients were exposed to the treatment sounds for longer periods.

We first identified more significant efficacy in the DFCRS treatment group compared to that in the UM control group during the treatment program by using linear mixed models. We further analyzed the possible influencing factors according to the different outcomes of tinnitus treatment and observed that those who achieved better therapeutic outcomes tended to adhere to acoustic treatment longer than those who reported aggravated tinnitus (Figure 2). Additionally, as shown in Figure 3, the assessment scores continued to decrease as the treatment proceeded and the total treatment time accumulated, indicating a steady increase in the efficacy of DFCRS. Interestingly, these 2 results on the treatment duration provided evidence from 2 perspectives for a possible positive correlation between the efficacy of DFCRS and treatment duration and were comparable to those reported in a previous 18-month controlled clinical trial study by Bauer and Brozoski [14] and an 18-month randomized controlled trial study by Henry et al [18], which used tinnitus retraining therapy or tinnitus masking sound.

THI, as the primary outcome, showed the treatment effects of functional, emotional, and catastrophic feedback on tinnitus [37,43]. The results showed significant improvement in the first month with a sustained effect in the following 2 months, indicating that the functional impairment caused by tinnitus and the patient’s negative emotional state rapidly improved after starting DFCRS treatment. Additional assessments, including HADS-A, HADS-D, AIS, FTQ, and TCS, also showed rapid and sustained efficacy after 3 months of DFCRS treatment, and the results demonstrated effective improvements in different aspects such as anxiety and depression, sleep disturbance, and other negative emotions related to tinnitus [41,42,44]. We hypothesized that the patient’s trust in the treatment strategy gradually decreased the negative emotions such as fear and pain after personalized acoustic treatment and that physiological abnormalities such as sleep disturbances could be resolved [45]. The negative emotional and physiological responses caused by tinnitus and tinnitus itself interact synergistically. The patients benefited from individualized sound therapy, which helped them develop a positive mindset to overcome tinnitus, and the relief of tinnitus, in return, boosted their confidence in conquering tinnitus, which formed a positive feedback loop [12]. This could explain why patients showed significant relief at the first follow-up visit since the start of the experiment, and the efficacy persisted at the next 2 follow-up visits and was long-lasting. The sample size in our study was larger than those in other similar clinical trials [44], and the participants adhered well to the DFCRS strategy, which guaranteed data credibility.

The main limitation of our study, similar to those of other early clinical trials, is the lack of randomized subgroups [15]. In randomized controlled trials, to avoid selective bias, a more validated evaluation of the effect of sound treatment can be obtained compared to that in nonrandomized controlled trials [18]. In fact, a randomized controlled trial of this sound treatment strategy is already underway based on the initial positive results from this trial [32]. In future trials, a longer follow-up period would be necessary to determine whether the efficacy would be more significant after a longer treatment period [15,45]. In addition, the maintenance of efficacy after stopping the intervention also needs to be explored in a longer trial [15].

In conclusion, we have confirmed the efficacy of DFCRS in treating patients with subjective tinnitus, and thus, it could be a promising treatment option for chronic subjective tinnitus. However, future research needs to be conducted in a more controlled setting, even with a randomized controlled trial, to draw a more precise conclusion.

## Appendix

Multimedia Appendix 1 TREND (Transparent Reporting of Evaluations with Nonrandomized Designs) statement checklist for nonrandomized control trials.

Multimedia Appendix 2 Schematic diagram of the modified strategy for digital frequency customized relieving sound.

Multimedia Appendix 3 Additional tables.

## Abbreviations

AIS: Athens Insomnia Scale
DFCRS: digital frequency customized relieving sound
FTQ: Fear of Tinnitus Questionnaire
FTRS: Fudan Tinnitus Relieving System
HADS: Hospital Anxiety and Depression Scale
HADS-A: Hospital Anxiety and Depression Scale-subscale anxiety
HADS-D: Hospital Anxiety and Depression Scale-subscale depression
OR: odds ratio
QoL: quality of life
TCS: Tinnitus Catastrophizing Scale
THI: Tinnitus Handicapped Inventory
TREND: Transparent Reporting of Evaluations with Nonrandomized Designs
UM: unmodified music

## Data Availability

The datasets generated and analyzed during this study are available from the corresponding author (DT) upon reasonable request.
