# Supplementary material for: Digital Frequency Customized Relieving Sound for Chronic Subjective Tinnitus Management: Prospective Controlled Study
Source: J Med Internet Res. 2025 Jan 17;27:e60150. doi: 10.2196/60150 (PMC11786133; doi:10.2196/60150)
Supplement: Multimedia Appendix 2 [file jmir_v27i1e60150_app2.docx]

Multimedia Appendix 2.


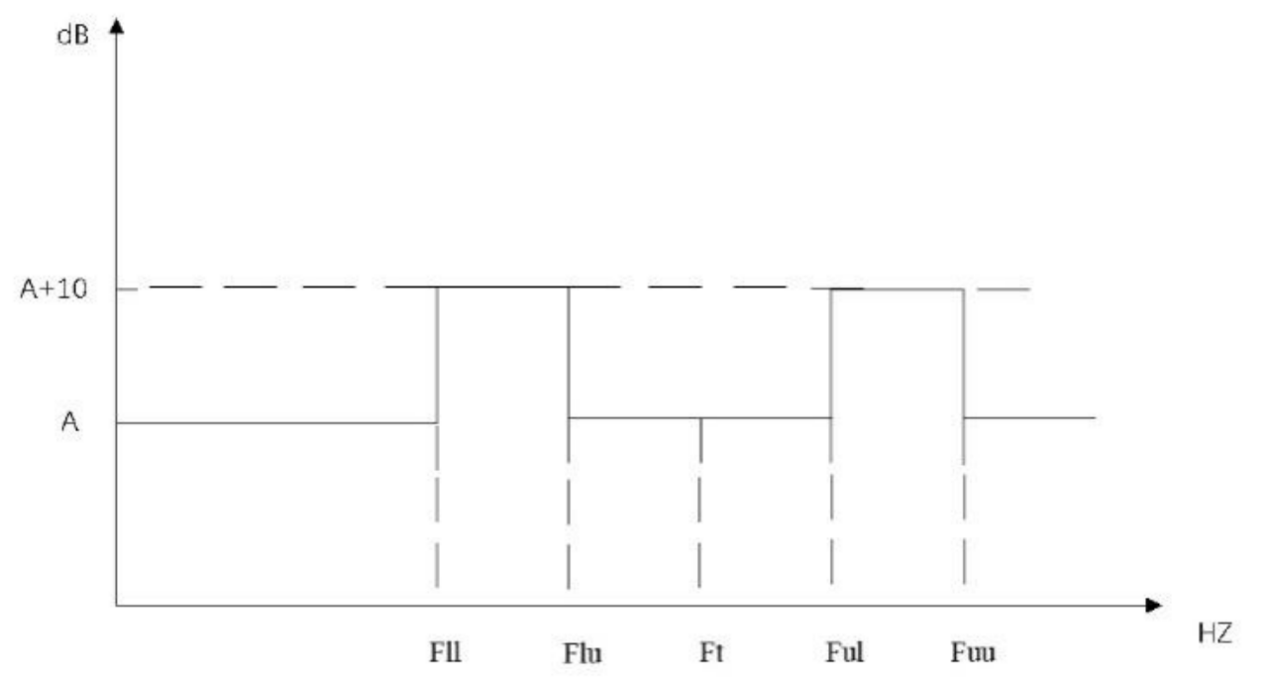


Figure S1. Schematic diagram of the modified strategy for DFCRS.

The formula transformation between $F_{t}$, $F_{l}$, and $F_{u}$ is as follows.

$$\begin{aligned} F_{u}=2F_{l}\#\left( AUTONUM \backslash* Arabic \right) \end{aligned}$$

$$\begin{aligned} F_{u}\times F_{l}=F_{t}^{2}\#\left( AUTONUM \backslash* Arabic \right) \end{aligned}$$

According to the definition of the 1/3 octave, the upper ($F_{uu}$) and lower ($F_{ul}$) end points of the 1/3 octave centered at $F_{u}$ are:

$$\begin{aligned} F_{uu}=(2^{\frac{1}{3}})^{\frac{1}{2}}\times F_{u}\#\left( AUTONUM \backslash* Arabic \right) \end{aligned}$$

$$\begin{aligned} F_{ul}=(2^{\frac{1}{3}})^{-\frac{1}{2}}\times Fu\#\left( AUTONUM \backslash* Arabic \right) \end{aligned}$$

The upper ($F_{lu}$) and lower ($F_{ll}$) end points of the 1/N octave centered at $F_{l}$ are:

$$\begin{aligned} F_{lu}=(2^{\frac{1}{3}})^{\frac{1}{2}}\times F_{l}\#\left( AUTONUM \backslash* Arabic \right) \end{aligned}$$

$$\begin{aligned} F_{ll}=(2^{\frac{1}{3}})^{-\frac{1}{2}}\times F_{l}\#\left( AUTONUM \backslash* Arabic \right) \end{aligned}$$
