# Supplementary material for: Digital Frequency Customized Relieving Sound for Chronic Subjective Tinnitus Management: Prospective Controlled Study
Source: J Med Internet Res. 2025 Jan 17;27:e60150. doi: 10.2196/60150 (PMC11786133; doi:10.2196/60150)
Supplement: Multimedia Appendix 3 [file jmir_v27i1e60150_app3.docx]

Multimedia Appendix 3.

Table S1. The trend results of multi-dimensional scales at baseline and three post- treatment follow-up visits in UM group.

| UM | Baseline  N=77 | 1 month  N=77 | 2 month  N=77 | 3 month  N=77 | p.overall | p.baseline vs 1 m | p.baseline vs 2 m | p.baseline vs 3 m | p.1 m vs 2 m | p.1 m vs 3 m | p.2 m vs 3 m |
| --- | --- | --- | --- | --- | --- | --- | --- | --- | --- | --- | --- |
| THI | 40.0 [24.0;60.0] | 43.0 [27.0;68.0] | 50.0 [33.0;79.0] | 49.0 [33.0;75.0] | 0.081 | 0.380 | 0.105 | 0.105 | 0.380 | 0.398 | 0.889 |
| HADSA | 5.00 [2.00;7.00] | 4.00 [2.00;7.00] | 4.00 [2.00;8.00] | 4.00 [2.00;8.00] | 0.969 | 0.972 | 0.972 | 0.972 | 0.972 | 0.972 | 0.972 |
| HADSD | 4.00 [1.00;7.00] | 4.00 [1.00;7.00] | 4.00 [1.00;8.00] | 3.00 [1.00;8.00] | 0.769 | 0.805 | 0.805 | 0.805 | 0.805 | 0.805 | 0.805 |
| AIS | 6.00 [4.00;9.00] | 7.00 [4.00;9.00] | 7.00 [4.00;9.00] | 7.00 [4.00;9.00] | 0.751 | 0.893 | 0.893 | 0.893 | 0.893 | 0.949 | 0.893 |
| VAS | 5.00 [3.00;6.00] | 5.00 [3.00;7.00] | 5.00 [3.00;8.00] | 4.00 [2.00;7.00] | 0.449 | 0.647 | 0.544 | 0.813 | 0.647 | 0.647 | 0.544 |
| FTQ | 6.00 [4.00;10.0] | 6.00 [3.00;11.0] | 7.00 [3.00;12.0] | 6.00 [3.00;11.0] | 0.847 | 0.905 | 0.905 | 0.942 | 0.905 | 0.905 | 0.905 |
| TCS | 22.0 [16.5;30.5] | 24.0 [18.0;34.0] | 28.0 [20.0;35.0] | 27.0 [20.0;35.0] | 0.056 | 0.395 | 0.072 | 0.104 | 0.330 | 0.395 | 0.613 |

Table S2. Demographic and tinnitus characteristics of DFCRS-treated participants divided into three treatment outcome groups and the group comparisons.

|  |  | Tinnitus worsening (n=8) | Tinnitus relief  (n=93) | Tinnitus disappearing (n=6) | *p* |
| --- | --- | --- | --- | --- | --- |
| gender (%) | male | 4 (50.0) | 47 (50.5) | 3 (50.0) | 0.999 |
|  | female | 4 (50.0) | 46 (49.5) | 3 (50.0) |  |
| location (%) | unilateral | 1 (12.5) | 35 (37.6) | 4 (66.7) | 0.219 |
|  | in brain | 0 (0.0) | 7 (7.5) | 0 (0.0) |  |
|  | bilateral | 7 (87.5) | 51 (54.8) | 2 (33.3) |  |
| tone (%) | unknown | 1 (12.5) | 32 (34.4) | 1 (16.7) | 0.089 |
|  | low-pitched | 0 (0.0) | 6 (6.5) | 1 (16.7) |  |
|  | high-pitched | 5 (62.5) | 52 (55.9) | 4 (66.7) |  |
|  | murmur | 2 (25.0) | 3 (3.2) | 0 (0.0) |  |
| severity (%) | unknown | 1 (12.5) | 28 (30.1) | 0 (0.0) | 0.629 |
|  | Ⅰ | 2 (25.0) | 10 (10.8) | 1 (16.7) |  |
|  | Ⅱ | 2 (25.0) | 14 (15.1) | 1 (16.7) |  |
|  | Ⅲ | 2 (25.0) | 29 (31.2) | 2 (33.3) |  |
|  | Ⅳ | 1 (12.5) | 12 (12.9) | 2 (33.3) |  |
| age | | 54.62 ± 13.20 | 45.25 ± 13.89 | 53.50 ± 8.46 | 0.079 |
| treat time (hours/day) | | 0.80 [0.50, 1.00] | 1.50 [1.00, 2.00] | 2.00 [1.62, 2.38] | 0.007 |
| tinnitus course (months) | | 30.00 [4.00, 60.00] | 12.00 [4.00, 24.00] | 2.50 [2.00, 4.50] | 0.069 |
| THI |  | 35.00 [29.00, 39.00] | 48.00 [36.00, 66.00] | 59.00 [51.50, 71.00] | 0.065 |
| HADS-A | | 4.00 [1.75, 6.25] | 5.00 [2.00, 8.00] | 8.50 [5.50, 10.75] | 0.212 |
| HADS-D | | 4.50 [3.00, 5.00] | 4.00 [2.00, 7.00] | 5.00 [3.25, 9.00] | 0.61 |
| AIS |  | 5.50 [4.00, 7.75] | 7.00 [4.00, 10.00] | 6.50 [3.25, 12.75] | 0.69 |
| FTQ |  | 6.50 [5.75, 8.75] | 7.00 [5.00, 10.00] | 8.00 [4.00, 10.50] | 0.938 |
| TCS |  | 22.62 ± 8.18 | 24.37 ± 9.63 | 27.00 ± 16.22 | 0.718 |
| left ear frequency (Hz) | | 8000 [8000, 8000] | 6350 [6350, 8000] |  | 0.347 |
| right ear frequency (Hz) | | 4000 [4000, 4000] | 6350[4520, 8000] | 6520 [5780, 7260] | 0.587 |
